# Complete Genome Sequence of the Endosymbiont of *Acanthamoeba* Strain UWC8, an Amoeba Endosymbiont Belonging to the “*Candidatus* Midichloriaceae” Family in *Rickettsiales*

**DOI:** 10.1128/genomeA.00791-14

**Published:** 2014-08-07

**Authors:** Zhang Wang, Martin Wu

**Affiliations:** Department of Biology, University of Virginia, Charlottesville, Virginia, USA

## Abstract

The endosymbiont of *Acanthamoeba* strain UWC8 is an obligate amoeba endosymbiont belonging to the family of “*Candidatus* Midichloriaceae” in *Rickettsiales.* We report here the complete genome sequence of this bacterium, which should catalyze future studies of amoeba-symbiont interactions.

## GENOME ANNOUNCEMENT

“*Candidatus* Midichloriaceae” is a recently defined family in *Rickettsiales* that is distinct from previously described *Rickettsiaceae*, *Anaplasmataceae*, and *Holosporaceae* families (1). It consists of intracellular bacteria residing within a wide range of host species, including ticks, fleas, stink bugs, amoebae, cnidarian, sponges, fish, and humans (2–11). “*Ca.* Midichloriaceae” is closely related to mitochondria (3). Despite being of significant ecological and evolutionary interest, only “*Candidatus* Midichloria mitochondrii” in this family has been sequenced (3). Here we report the complete genome sequence of the endosymbiont of *Acanthamoeba* UWC8 that belongs to the “*Ca.* Midichloriaceae” family.

The *Acanthamoeba* UWC8 strain harboring the endosymbiont was obtained from ATCC and cultured according to ATCC’s protocol. The amoeba cells from 10 T-75 tissue flasks were harvested and resuspended in the endosymbiont extraction buffer (0.3 M-sucrose, 10 mM Tris-HCl, 5 mM MgCl_2_, pH 7.5). The cells were subsequently homogenized using a Dounce tissue grinder (Wheaton), and endosymbiont cells were isolated by differential centrifugation. The genome was sequenced by a 454 GS-FLX System using a 3-kb paired-end library, generating 104,222 reads totaling 31 Mbp with an average coverage depth of 20×. The genome was assembled by GS *De Novo* Assembler (Newbler) version 2.5.3. PCR and Sanger sequencing were used to close the gaps between contigs when necessary.

The complete genome of the endosymbiont of *Acanthamoeba* UWC8 consists of a single molecule of 1,615,277 bp with a G+C content of 34.7%. The genome was annotated using the IGS Annotation Engine (12): 89.5% of the genome was predicted to encode 1,608 open reading frames (ORFs), 36 tRNA genes, and one rRNA operon, and, of these, 1,049 (65.2%) ORFs can be assigned a putative function. The genome is predicted to encode major metabolic pathways, including a complete TCA cycle, electron transport chain, and pentose phosphate pathway. While most enzymes of glycolysis are present in the genome, the key enzyme phosphofructokinase (*PfkA*) converting beta-D-fructose-6P to beta-D-fructose-1,6-2P is missing. Instead, the fructose-1,6-bisphosphatase (*fbp*) catalyzing the opposite reaction is encoded in the genome, suggesting that gluconeogenesis instead of glycolysis is functional. Consistent with its obligate intracellular lifestyle, the endosymbiont is deficient in the biosynthesis of many of its own metabolic substances, including amino acids, nucleotides, and several coenzymes. Accordingly, it encodes a large number of ABC-type transporters and solute carrier families importing most of these nutrients from the host. The genome encodes complete pathways for synthesizing both peptidoglycan and lipopolysaccharide (LPS), suggesting that a complete cell envelope structure can be assembled. In addition, the genome encodes 4 genes in capsule biosynthesis and secretion that are absent in other *Rickettsiales*, which is consistent with the previous finding that capsules or slime layers are produced by this bacterium (13). This genome represents a valuable resource for future studies of this organism and amoeba-symbiont interactions in general.

### Nucleotide sequence accession number.

The complete genome sequence of the endosymbiont of *Acanthamoeba* UWC8 has been deposited at DDBJ/EMBL/GenBank under the accession number CP004403.
